# Using art to raise awareness of breastfed children with medical complexity

**DOI:** 10.1186/s13006-022-00488-3

**Published:** 2022-06-27

**Authors:** Lyndsey Hookway

**Affiliations:** School of Health and Social Care, Swansea University, Swansea, Wales UK

**Keywords:** Breastfeeding, Paediatrics, Art, Healthcare professionals

## Abstract

**Background:**

Current infant feeding support is often targeted at establishing breastfeeding in healthy term infants, or supporting lactation for preterm infants in the neonatal setting. Breastfeeding presents different challenges for children beyond the neonatal period who have a medical complexity. The tendency to focus on breastfeeding as a preventative public health intervention overlooks the fact that mothers and children with medical complexity often require additional and targeted support to continue to breastfeed. Despite this identified need, there is very little research, policy or clinically specific teaching within paediatrics that is nuanced enough to support this vulnerable population.

**Raising awareness of breastfed children with medical complexity in paediatrics:**

While research, policy and embedded Baby Friendly Health Initiative (BFHI) standards in both the maternity and neonatal directorates exists, paediatrics is a separate discipline and contrastingly, has very little lactation support infrastructure. To this end, a doctoral study was commenced aiming to identify the differences for this vulnerable and largely overlooked group. One of the related outputs of the ongoing research is the use of creative methods to raise awareness. This commentary highlights a project with an artist to develop a series of portraits alongside a book and health professional education to increase awareness of these children’s needs.

**Conclusions:**

The breastfeeding needs and challenges of children in the paediatric setting are unique and require additional awareness, creativity and skills to support optimal infant and young child breastfeeding. Using art can connect professionals to the lived experiences of families trying to persevere through medical complexity.

## Background

Optimising breastfeeding may be particularly beneficial for infants and children with complex medical needs to prevent additional disease and infection [1–4], as well as to provide comfort [5] and pain relief [6, 7].

However, childhood acute and chronic illness or disability is associated with increased challenge and lower breastfeeding rates [8–11]. Many of these sick children are cared for in the paediatric setting, which is a separate organisational directorate to the neonatal and maternity units. The Baby Friendly Hospital Initiative (BFHI) has been shown to increase initiation of breastfeeding [12]. However, the standards have not been adapted for clinical use with older children or children with complex needs, and BFHI is also not widely adopted in paediatric wards or hospitals. This means that there is usually not a designated lactation support service on paediatric wards and no mandatory training or audit for paediatric clinicians. While expertise is sometimes ‘borrowed’ from maternity or neonatal colleagues, this is dependent on workload and staff availability and is thus only inconsistently provided [13].

Therefore, in paediatrics, not only do mothers of children with medical complexity have different challenges initiating and maintaining breastfeeding compared to healthy newborns or preterm neonates, but they also receive less support to overcome these challenges.

The needs of breastfed children and their mothers in paediatrics is under-researched. There is therefore a paucity of literature about this population, and the literature that exists is often dated, and focused on specific conditions – namely cardiac conditions [14–17], cleft lip and palate [17, 18], Down syndrome [19, 20], and phenylketonuria [21]. At present, there is very limited literature relating more generally to supporting children with various medical challenges beyond the neonatal period.

While much is known about what works to support breastfeeding initiation, duration and exclusivity [22], many of the systemic structures that enable optimal breastfeeding are not in place on the paediatric ward.

As a paediatric nurse, International Board Certified Lactation Consultant (IBCLC), and mother of a breastfed childhood cancer and sepsis survivor, the frustrations with widespread lack of breastfeeding support for families within paediatrics led me to commence a doctoral study. This study aims to understand the challenges for children with medical complexity, as well as the gaps in knowledge for the health and lactation professionals supporting them. Alongside the PhD, a support group – *Breastfeeding the Brave* – hosted in Facebook, was established to provide support and advocacy for mothers and parents breastfeeding their children with various medical needs. Different strategies have been used to educate and raise awareness, including the use of social media and video. A project utilising art is the latest innovation to promote better understanding of this under-studied area. Alongside the researcher, Leanne Pearce, an artist known for her art with purpose, chose several images shared by parents to capture in oil paint, in the hope that professionals will understand the research by connecting with the stories through the art. The portraits will be toured in an exhibition, used in teaching, digital prints will be available, and prints will also be donated to various hospitals. All parents have been keen to share their stories widely to support both healthcare professional learning and development, as well as inspire parents going through similar challenges, and all consented to being included in this article.

## Using stories and art to augment research impact

Many healthcare professionals, academics, policymakers and even non-medical lactation professionals do not universally understand the specific differences for this population of children with complex medical needs. The more familiar research and lactation strategies within the maternity and neonatal settings may not always apply to this group but, without clinical experience, professionals may struggle to appreciate the challenges. Health and lactation professionals are thus welcomed into the Breastfeeding the Brave group in order to passively learn about the challenges of breastfeeding children with medical complexity in a unique way. However, more education is needed, and therefore creative approaches to raising the profile of this group have been utilised.

One way to support professional learning is to use art and stories to connect new and emerging theory to lived experiences. Art in healthcare teaching is not a commonly utilised approach, despite caring often being described as an ‘art’ and the fact that it has been shown to help practitioners understand caring [23] and become more compassionate [24]. Art can be used successfully in healthcare teaching [25] and has been shown to support professionals to better understand the patient perspective, as well as enhance communication and empathy [26]. One study found that students exposed to art-based teaching were better able to connect the empathic and cognitive aspects of learning [27]. Art can be used therapeutically both to support suffering and grieving parents, but it has also been shown to reduce compassion fatigue in healthcare professionals [28].

## A unique population

Breastfeeding may be harder for mothers of children with medical needs for a variety of reasons. A recent systematic review found seven key themes in the available literature [29]. Broadly, there were four parent- and child-related themes, and three professional and institutional themes. Parent themes included logistical and practical problems of being resident overnight, the general unwillingness by staff to facilitate bedsharing, difficulties accessing usual community breastfeeding support and practical breastfeeding challenges such as mastitis and low supply that were not adequately managed in the paediatric setting.

**Fig. 1:**
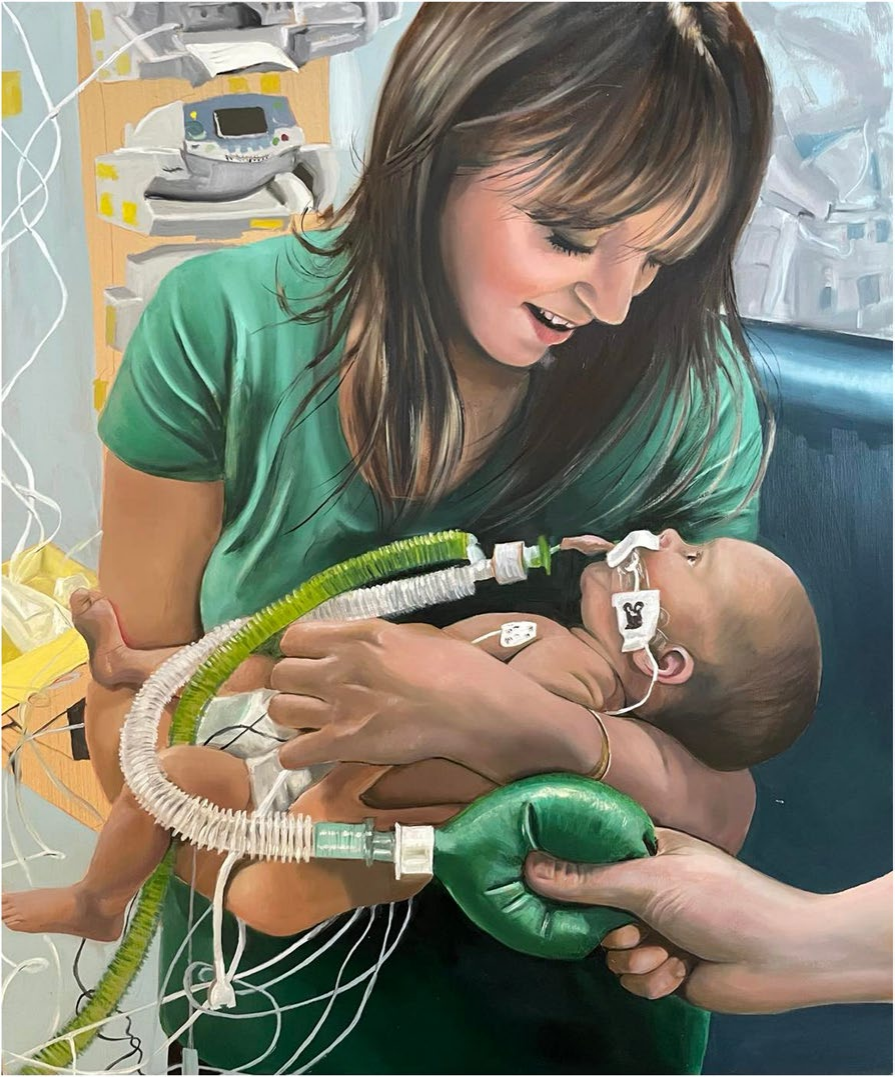
Hannah and Maisie. Hannah is holding Maisie while supported by clinical staff in the PICU who were continuing to provide critical care. *PICU* paediatric intensive care unit. Artist: Leanne Pearce

Mothers also struggled with psychological challenges, with many reporting anxiety, depression, stress and impaired milk ejection reflex due to the pressure of needing to produce high volumes under extraordinarily stressful circumstances. Numerous parents in the Breastfeeding the Brave group have reported that these issues continue to be problematic, including one mother of a child born with mitochondrial disease. The child spent much of her life in hospital and sadly died just before she was 6 months old. Throughout her daughter’s life, her mother had to defend feeding for comfort, skin to skin and bedsharing (Fig. 1).

When parents in the group have endured trauma and grief that has been made more difficult by lack of understanding, they have often been highly motivated to get involved in projects that seek to address the encountered challenges. In this way, the art benefits both the subject and the viewer.

## The challenge of conveying nuance

While aspects of breastfeeding support in different settings overlap, different skills are sometimes needed to support lactation in older sick children. Since hospitalised families often find it impractical to access sources of community support, the burden of responsibility lies with clinical staff to support lactation alongside critical care which is difficult if they have received no training – a common finding [29–34].

Current training and knowledge usually leans towards the challenges most commonly encountered by staff supporting breastfeeding in the maternity and neonatal settings. Therefore, art may be an innovative way to convey the nuances, visually connecting the healthcare professional with the lived experience of older children. In one study, the researchers noted that art may increase healthcare staff awareness of suffering, and thus improve nursing care. However, they also note that observation of art activates reward centres in the brain which makes associated learning pleasurable and thus more memorable [35]. This has potential implications for improving not only awareness but also retention of new learning.

It is hoped that the paintings will illustrate several key differences and problems in the paediatric setting. For example, there are multiple research studies supporting the use of human milk for preterm neonates to promote optimal neurodevelopment and reduction in risk of necrotising enterocolitis [36–38] thus, early expressing and the use of donor human milk is more likely to be expected and encouraged. While it is recognised that positive experiences of receiving support in the neonatal unit are not universal, by comparison, paediatrics lags behind. No studies discuss scaling up of donor human milk provision within paediatrics, nor are there studies that explore skin to skin care within paediatric wards or the paediatric intensive care unit (PICU), compared with the many studies that support this intervention in neonatal units [39–42]. When no literature exists, it is difficult to recommend or defend practice change, even in very difficult or palliative cases where it would be a compassionate intervention to improve quality of life. Art and stories that depict these aspects of unresearched care may increase the prevalence of these interventions on the paediatric ward.

**Fig. 2:**
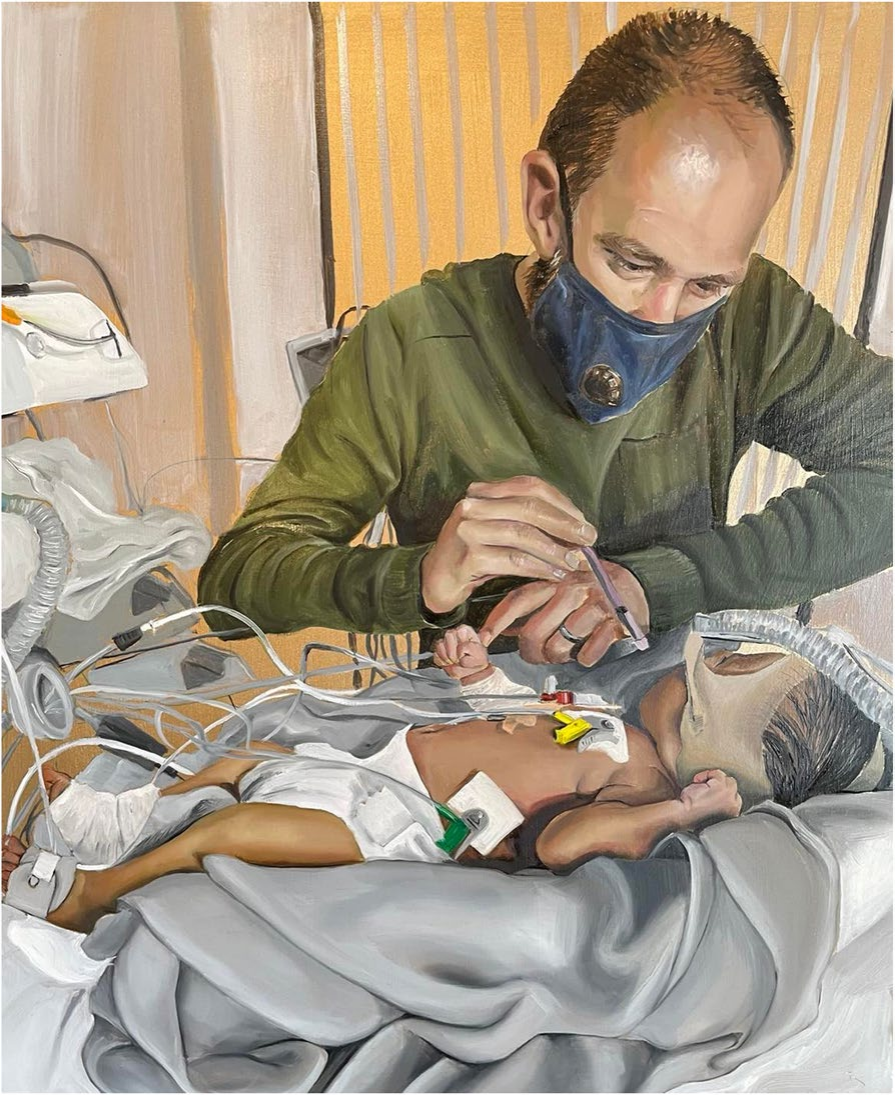
Edward and Jacob. Jacob’s father Edward is feeding his son Jacob with Laura’s expressed milk via a syringe while he was in the HDU. *HDU* high dependency unit. Artist: Leanne Pearce

Practically speaking, it can be awkward to breastfeed children with lines, drains, large wounds, stomas, splints, and casts, as well as those receiving non-invasive respiratory support, and larger children can be difficult to manoeuvre into a mutually comfortable position. Difficulties establishing oral feeding after surgery have been found to be more common with longer ventilation times [43]. Maintaining milk supply and returning to breastfeeding is not always straightforward after enteral feeding, supplementation, suctioning, opiate withdrawal or ventilation. One baby had an episode of prolonged apnoea and became unresponsive at home. He was treated in the paediatric high dependency unit (HDU) and his mother was given no information about how to maintain her milk supply. The mother used her own breast pump but most of the staff were unaware or unsupportive of using expressed breastmilk. Maintaining milk supply was extremely challenging but the mother persevered largely through her own knowledge and experience (Fig. 2).

Supporting breastfed children who are unwell or have medically complex conditions and needs is a less well-trodden path. Paediatrics can feel like a breastfeeding ‘skill-desert’, with families cared for in a clinical environment that is not able to fully support their feeding goals. Both healthcare and lactation staff can only develop increased awareness about these challenges and how to overcome them once they are cognisant of them. Art might be one channel through which we can prompt curiosity and problem-solving.

## How art may support understanding and improve patient experience

Patient stories are meaningful, and for practitioners who are unfamiliar with a novel topic, art depicting these stories can provide another way of learning [44]. Art has been shown to improve psychological wellbeing [35]. This is sometimes because the person observing the art finds pleasure in looking at it, but enjoyment can also be derived by understanding the meaning of the artwork [35]. Indeed, it has also been suggested that upsetting subject matter can also provoke a positive response, which is why tragedy is a popular genre [45]. Negative emotions can create a high level of emotional response and make the subject matter more memorable, which is particularly pertinent for staff supporting breastfed children with medical complexity [45].

In other healthcare settings where artwork is displayed, it can transform a clinical environment into a space that celebrates the bravery of individuals enduring health challenges. This publicly displayed courage serves to inspire both fellow patients and families, and also staff [46].

One study found that art can become an important part of the psychological needs of paediatric patients and their families [47]. Healthcare professionals often report positive impacts of interventions such as art therapy for children, and art can introduce a concept in a non-threatening way. Thus, having images displayed may serve to open conversations or provide a passive sense of permission or possibility for breastfeeding in a situation where it might otherwise feel impossible. A further benefit of art is that healthcare professionals often deal with traumatic and distressing experiences during the course of their working day. The needs of professionals who support dying children and their bereaved families are often unmet. Art may be potentially one way for healthcare staff to acknowledge this more difficult aspect of their role [47].

Finally, one study found that teaching medical students using artwork can help them connect visual clues to underlying disease. The skill of observation can be taught, and art may serve as a medium through which we can teach healthcare professionals not only to care, but to accurately observe both the obvious and the subtler clues [48].

**Fig. 3:**
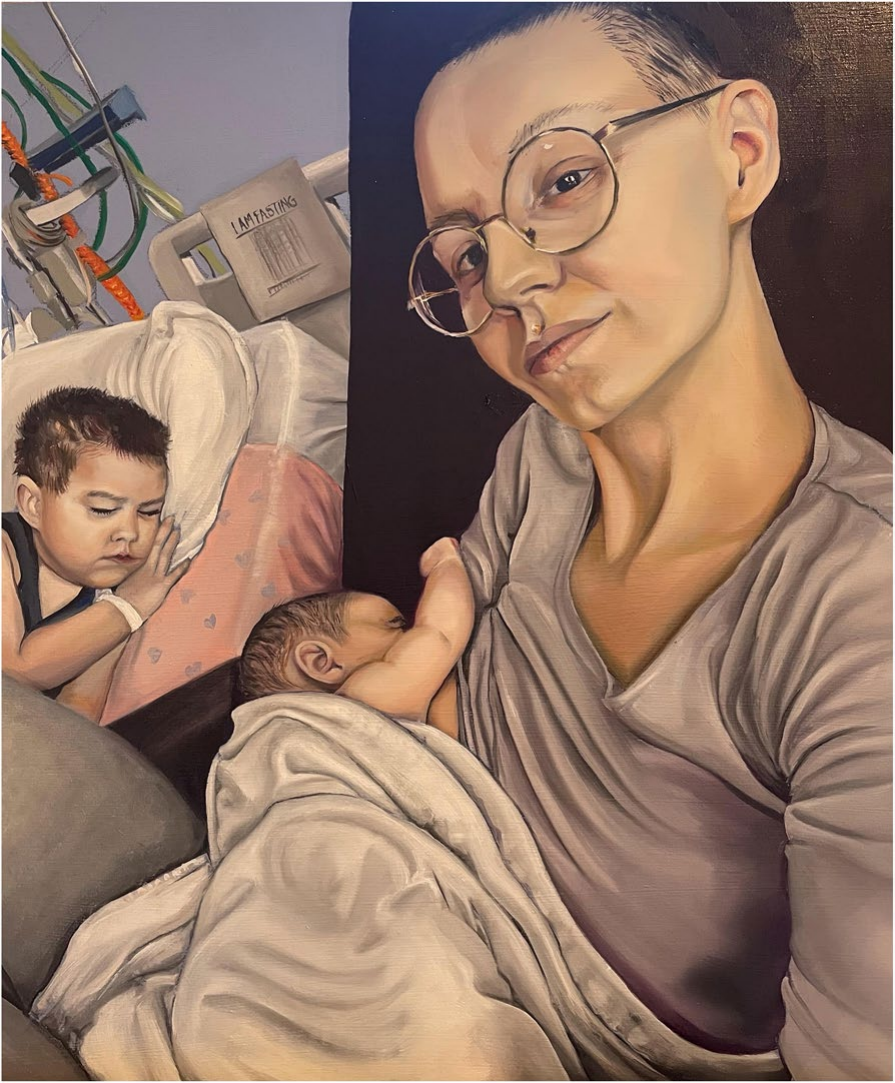
Cara with her two unwell children. Cara is breastfeeding 4-month old Maya at the hospital bedside of Jayke, aged four. Artist: Leanne Pearce

Through the artwork being created, we invite viewers to see a family’s perspective through the combination of their story and portrait. Some of the portraits convey tenderness and connection in the face of adversity (Fig. 1) and others hint at the practical challenges of maintaining breastfeeding (Fig. 2). Some portraits were chosen to illustrate strength and determination (Fig. 3).

The older child in Fig. 3 was diagnosed with acute lymphoblastic leukaemia at the age of 4 years. His infant sister was then diagnosed with Fibromatosis requiring chemotherapy 4 months later. The children’s mother was exclusively breastfeeding her daughter, and in addition expressed milk for her son, fed via his nasogastric tube when he was undergoing intensive chemotherapy. The mother recalls that one of the hardest parts was caring for two sick children away from home. She and her husband would take it in turns to stay in hospital accommodation while the other was resident for long periods on the paediatric oncology ward. The practical challenge of trying to maintain normal parenting routines and continue to breastfeed in an unfamiliar and intense environment, with serious health challenges was significant.

While all the stories the parents have to tell are profound, the art that accompanies them allows the reader an additional insight into the private struggle. In all the images, re-created from photos shared by the parents, a window into the world of breastfeeding a child with medical complexity is opened. The artwork alone cannot teach professionals how to manage similar situations, yet it may provide opportunities to discuss clinical scenarios where creative adaptations and novel approaches might be required. More importantly, the portraits provide inspiration, reassurance and hope to both professionals and parents that despite huge challenges, breastfeeding *is* possible.

## Conclusion

In an era of evidence-based practice and an increasing importance for demonstrating empirical proof, research into under-studied areas is a priority. However, these are by definition usually topics about which people have very little knowledge or awareness. Writing and research in these cases may effectively convey a literary narrative of a novel concept or experience, but they may not connect the reader as effectively as visual imagery.

By using art that captures the poignancy of a moment, we hope to be able to more accurately and meaningfully convey the lived experiences of mothers and parents breastfeeding their sick children.

## Data Availability

Not applicable.

## Abbreviations

BFHI: Baby Friendly Hospital Initiative
HDU: High Dependency Unit
IBCLC: International Board Certified Lactation Consultant
PICU: Paediatric Intensive Care Unit
